# *In vitro* 3D modeling of colorectal cancer: the pivotal role of the extracellular matrix, stroma and immune modulation

**DOI:** 10.3389/fgene.2025.1545017

**Published:** 2025-05-01

**Authors:** Veroniaina Hanitrarimalala, Zdenka Prgomet, My Hedhammar, Helena Tassidis, Anette Gjörloff Wingren

**Affiliations:** ^1^ Department of Biomedical Sciences, Faculty of Health and Society, Malmö University, Malmö, Sweden; ^2^ Biofilms-Research Center for Biointerfaces, Malmö University, Malmö, Sweden; ^3^ KTH Royal Institute of Technology, Division of Protein Technology, Stockholm, Sweden; ^4^ Department of Bioanalysis, Faculty of Natural Sciences, Kristianstad University, Kristianstad, Sweden

**Keywords:** colorectal cancer, *in vitro*, 3D model, tumor microenvironment, extracellular matrix, immune cells, therapy

## Abstract

Colorectal cancer (CRC) is a leading global cancer with high mortality, especially in metastatic cases, with limited therapeutic options. The tumor microenvironment (TME), a network comprising various immune cells, stromal cells and extracellular (ECM) components plays a crucial role in influencing tumor progression and therapy outcome. The genetic heterogeneity of CRC and the complex TME complicates the development of effective, personalized treatment strategies. The prognosis has slowly improved during the past decades, but metastatic CRC (mCRC) is common among patients and is still associated with low survival. The therapeutic options for CRC differ from those for mCRC and include surgery (mostly for CRC), chemotherapy, growth factor receptor signaling pathway targeting, as well as immunotherapy. Malignant CRC cells are established in the TME, which varies depending on the primary or metastatic site. Herein, we review the role and interactions of several ECM components in 3D models of CRC and mCRC tumor cells, with an emphasis on how the TME affects tumor growth and treatment. This comprehensive summary provides support for the development of 3D models that mimic the interactions within the TME, which will be essential for the development of novel anticancer therapies.

## 1 Introduction

**FIGURE 1:**
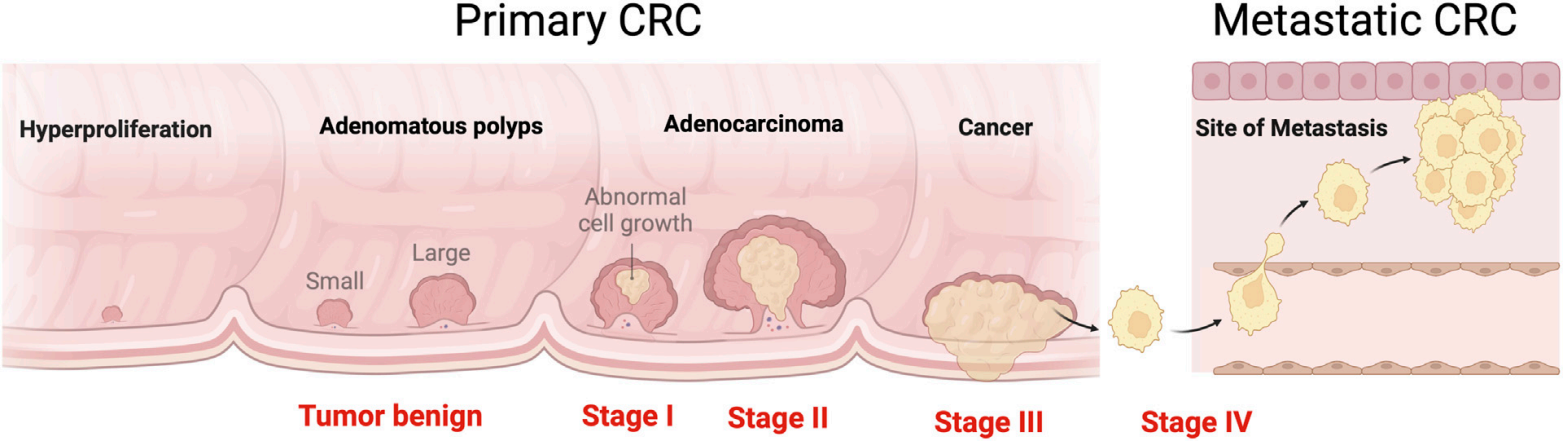
CRC development and transformation in different stages from adenomatous polyps to metastases. Four stages in the development of CRC carcinogenesis occur: initiation, promotion, progression, and metastasis. The most common metastatic site is the liver, followed by the lung and bone. Created with BioRender.com.

Colorectal cancer (CRC), which includes colon and/or rectum cancer, is a major public health concern because it is the third most diagnosed and second most lethal cancer worldwide (Morgan et al., 2023). CRC accounted for approximately 9% of all cancer-related deaths in 2022 (Bray et al., 2024), and the incidence of this disease is expected to rise to 2.5 million new cases by 2035 (Dekker et al., 2019). CRC is related to environmental factors (e.g., smoking, diet, obesity, alcohol), immune system dysregulation, or genetics, such as mutations of microsatellite instability (MSI) genes *MSH1, MLH1,* and *MSH6,* adenomatous polyposis coli *(APC),* or nucleotide-binding oligomerization domain 2 (*NOD2*) (Lawes et al., 2005). CRC begins with the transformation of a normal colonic crypt into a hyperproliferation and then into a benign adenomatous polyp (Figure 1). A small proportion of these adenomatous polyps will progress to advanced adenocarcinoma, causing cancerous growth (malignant neoplasia) and metastases. Progressive transition, also known as ‘multistep carcinogenesis’, occurs during CRC development, and unique genetic modifications of tumor suppressors or oncogenes correspond to each stage (Fearon, 2011). Mutations with the tumor suppressor genes like *APC* and *TP53* are usually associated with CRC (Alzahrani et al., 2021). Oncogenes involved in CRC include RAS genes (*KRAS*, *HRAS*, and *NRAS*), *BRAF*, *AKT1*, *EGFR*, *PIK3CA*, *MYC*, and *JAK* (Menyhart et al., 2019).

The overall 5-year survival rate for CRC patients is 65%, although the individual prognosis is largely determined by whether the patient develops metastases during the disease. Within 5 years, up to 60% of all CRC patients develop metastatic CRC (mCRC), resulting in a dismal survival rate of less than 15% (Siegel et al., 2023). Approximately 15% of CRC patients have liver metastases at the time of diagnosis (Manfredi et al., 2006), and an additional 16%–20% develop hepatic metastases within the first 3 years (Kemeny, 2007). Lung metastases are less common and mostly occur in patients with rectal cancer (Mitry et al., 2010). These data highlight the importance of more effective treatments for both CRC and mCRC.

**FIGURE 2:**
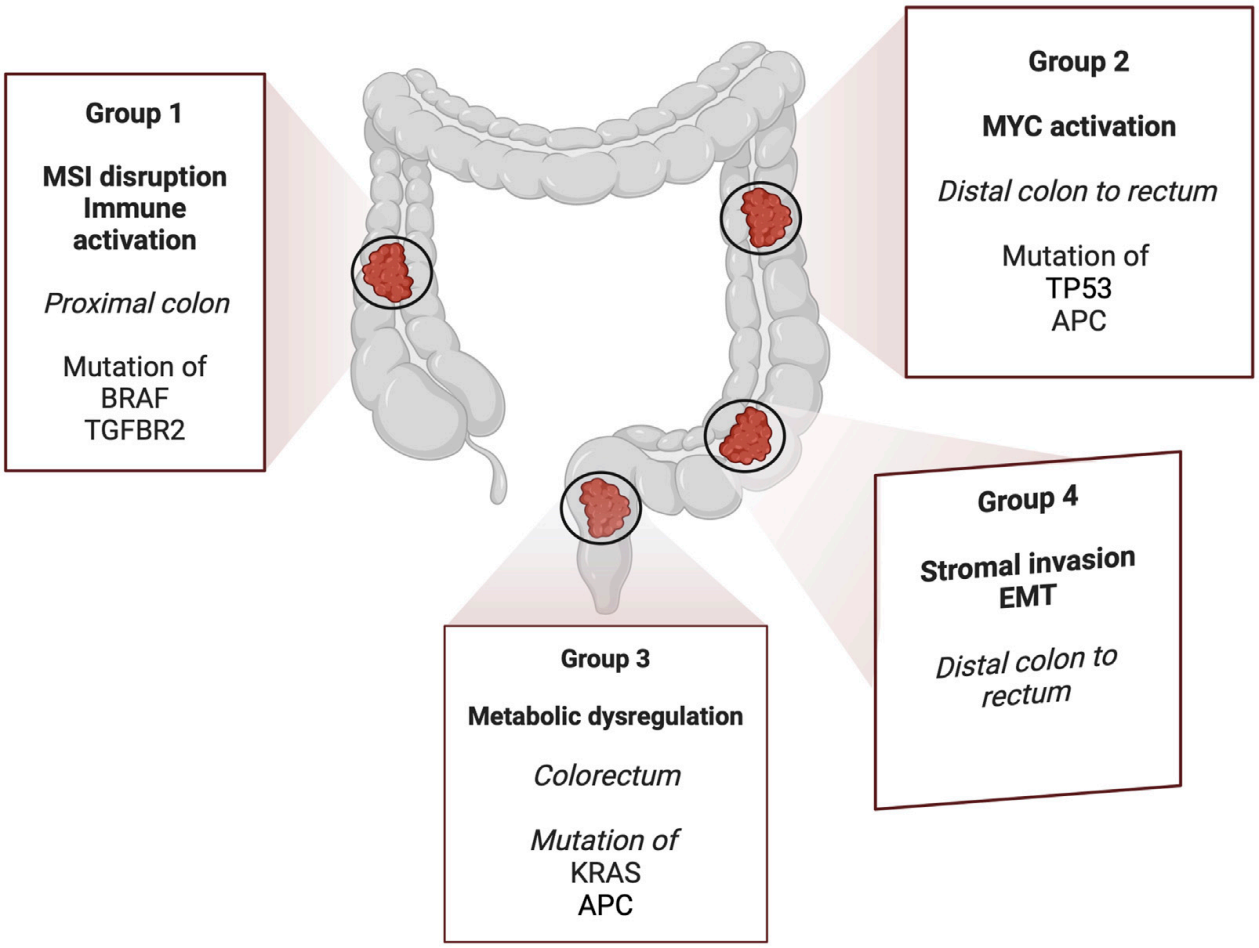
CRC are divided into four different groups based on the consensus molecular subtypes. The first group is characterized by MSI, high mutations of *BRAF* and activated immune components. The second involves epithelial differentiation and MYC mutations, causing high activity of these intracellular signaling pathways. It also features increased expression of EGFR, TP53 and APC. The third group shows metabolic dysregulation with increased activity in glutaminolysis and lipidogenesis, enriched with *KRAS* activating mutations. The fourth group show the expression of genes related to stromal invasion, epithelial–mesenchymal transition (EMT), transforming growth factor-beta (TGF-β), and angiogenesis. Created with BioRender.com.

Clinically, the CRC staging system defines tumor severity on the basis of histopathological features and helps estimate the probability of disease relapse in patients with locoregional CRC (stages I–III) and those diagnosed with metastasis (stage IV) (Cañellas-Socias et al., 2024). Owing to limits in early detection, a considerable majority of patients are in an advanced stage when they are diagnosed. The most common treatment strategy for early-stage CRC is surgery followed by chemotherapy or, in certain situations, radiotherapy in the rectum. CRC can also be divided into four groups based on its consensus molecular subtypes (Figure 2) (Guinney et al., 2015). The first group contains disrupted MSI and activated immune components. The second involves epithelial differentiation and MYC signaling activation. The third group shows metabolic dysregulation, while the fourth group shows the expression of genes related to stromal invasion, epithelial–mesenchymal transition (EMT), transforming growth factor-beta (TGF-β), and angiogenesis.

The introduction of 3D culture systems to *in vitro* modelling is expected to have a revolutionary impact on CRC research. With 3D culture options, it is possible to better replicate the human tumor and the complex tumor microenvironment (TME) of CRC, which is crucial in CRC development, progression, and metastasis. By mimicking the *in vivo* conditions of the tumor more closely by including microenvironment components such as stroma, immune cells and various extracellular matrix (ECM) glycoproteins, i.e., collagen, fibronectin, and laminins, 3D models offer significant advantages for the understanding of tumor biology, drug responses, and potential therapeutic strategies. A deep comprehension of the TME components is expected to allow development of effective CRC treatments. Recently, several 3D *in vitro* models have been developed specifically for CRC, including but not limited to spheroids, patient-derived organoids (PDO), bioprinted models, microfluidic models and scaffold-based models (Vitale et al., 2024; Li J. et al., 2024; Mo et al., 2022). 3D models became the most preferred approach in cancer research to fill the gap between “absolute *in vitro*” and “true *in vivo*” (Abbas et al., 2023). This review addresses the challenges of mimicking the TME components in an *in vitro* 3D model for CRC. It also underscores the necessity of developing more physiologically relevant 3D models. The importance of the role of the different ECM components in 3D models of CRC are discussed with an emphasis on how the TME affects tumor growth and treatment. Support is provided that there is a great need for *in vitro* 3D models that mimic the interactions within the TME, essential for screening for novel anticancer therapies. Furthermore, the knowledge of the TME and how nanotechnology can be integrated is highlighted. Therefore, a deep comprehension of the TME components is explored to find the better way for an effective CRC treatment. This review aims to bridge the gap between current model systems and the intricate biology of CRC.

## 2 Current *in vitro* models of CRC and mCRC

2D cell culture is one of the most common methods for studying CRC cells *in vitro*. However, these cell cultures involve the growth of a standardized monolayer, and some of the functionally important epithelial cell characteristics are not developed, potentially yielding results with little physiological value. For example, culturing several CRC epithelial cell types on an ultra-stiff plastic matrix induces cell proliferation and mesenchymal-like malignant phenotypes (Chaudhuri et al., 2014). Additionally, the lack of signals given by other stroma cell types may modify the morphological organization or response of intestinal cells (Paduch et al., 2010), or make sensitive tumor cell lines resistant to targeted drugs (Straussman et al., 2012). As a result, several analyses on cellular processes, such as proliferation, differentiation or apoptosis may be aberrant or not entirely true (Edmondson et al., 2014).

Spheroids are multicellular self-assembled 3D structures that can be generated via either scaffold-free or scaffold-based culture methods (Ramos et al., 2023). Scaffold-free spheroid formation methods include the hanging drop method, and culture using non-adherent surfaces (Reidy et al., 2021; Hasterok et al., 2023). Scaffold-free 3D cell cultures rely on the fact that cells adhere to each other when no other options are available. The hanging drop method uses surface tension and gravitational force to form spheroids by gathering cells in the lower part of a droplet hanging from an inverted tray (Jensen and Teng, 2020). Due to the ability of many adherent tumor cells to aggregate into higher cell densities, 3D spheroids have been widely used as cancer models *in vitro*.

The scaffold-based 3D culture methods typically use some type of hydrogel, and cell cultures can be achieved with either natural hydrogels (e.g., chitosan, alginate, silk fibroin) or synthetic hydrogels (e.g., polyethylene glycol (PEG), polylactic acid, and polyglycolic acid) (Ramos et al., 2023). Hydrogels are cross-linked networks formed of hydrophilic polymers attached through physical, electrostatic, or covalent interactions. Most hydrogels of synthetic polymers exhibit versatile biophysical, mechanical, and biological properties (Habanjar et al., 2021). However, synthetic polymers are unable to provide the biochemical signals necessary to “communicate” with the cell. To overcome this limitation, synthetic polymers can be functionalized by adding signalling biomolecules, such as peptides, growth factors, and glycans. Various natural polymers, of either animal or plant origin, can be used to form hydrogels. Examples of such natural polymers are animal-derived collagen, laminins, and fibrinogen, microbial-derived hyaluronic acid (HA) or plant-derived alginate and gellan gum (Bray et al., 2015). Collagen-based hydrogels are often used for 3D culture, since collagen is a major constituent of both the basement membrane and interstitial ECM. The commercially available Matrigel ^®^(Corning, Corning, NY, United States of America), is derived from mice with induced Engelberth–Holm–Swarm chondrosarcoma rich in ECM components. Those include laminin-111, type IV collagen, entactins and perlecan, as well as soluble growth factors, such as fibroblast growth factor (FGF), epidermal growth factor (EGF), transformative growth factor beta (TGF-beta), and MMPs (Marchini and Gelain, 2022). Since animal-derived ECMs substrates, such as collagen and Matrigel ^®^, are characterized by a poorly defined composition and batch-to-batch variability, increasing efforts have been made to develop bioinspired or fully-synthetic materials that could replace naturally-derived matrices, aiming for protocols that are more reproducible and translatable into clinical applications. For example, recombinant versions of ECM proteins such as laminins are commercially avalaible (BioLamina). Due to the more complicated post-translational modifications needed to obtain structurally functional collagen, the recombinant versions of fibrillar collagens are still difficult to obtain from recombinant processes (Fertala, 2020). Typical cell sources for CRC *in vitro* models are various immortalized cancer cell lines or dissociated cell clusters from resected tumors. There are a huge number of available primary CRC cell lines (Berg et al., 2017) whereof HCT116, HT29 and SW620 are commonly used for representation of the primary tumor. In contrast, established mCRC cell lines from the liver are less common, but exist (Boot et al., 2016).

In recent years, more and more investigations have been performed using spheroids formed from fresh primary cancer cells (Jeppesen et al., 2017) and patient-derived explants (Vitale et al., 2024). However, the typically limited life span (∼72 h) of primary cultures hinders longer studies of e.g., drug treatment effects. Lately, 3D culture into so called organoids have been more and more developed. Organoids refer to more complex cellular arrangements of organ-specific cells, that can be derived from pluripotent stem cells (PSCs), adult stem cells, or somatic cells of human tissues such as cancer cells (Ahmad Zawawi et al., 2024). Organoids mimic complex key structure, function, and biology of organs or tissues from which they are derived. The cells exhibit extended proliferation and differentiation capabilities over a long-term of culture, depending on specific culture medium used, and possess certain characteristics reminiscent of the organ or tumor from where they derived from. Pioneering development for organoid establishment was done by embedding isolated intestinal crypts or Lgr5-expressing intestinal stem cells into ECM and adding a special culture medium that contains essential growth factors, to obtain intestinal organoids (Sato et al., 2009). Since CRC, especially differentiated types that form ductal structures, are composed of a heterogeneous population of undifferentiated cancer stem cells (CSCs) and differentiated cells, constituting a hierarchical structure, a lot of efforts have been made to understand the role of CSCs in organoids from intestinal cells (Kawai et al., 2020). By the use of similar culture protocols, PDOs can be obtained from individual patients with high success rate, short culture period and unlimited expansion, which can highly recapitulate physiology of the original tumor (Guinney et al., 2015). Studies have shown that PDOs can not only be used to explore tumor biological characteristics in basic research,but also be used as a preclinical model to predict patients’ response to treatment (Mo et al., 2022). Indeed, CRC organoids retain tumor gene characteristics, heterogeneity, and intratumoral cellular heterogeneity, almost perfectly replicating CRC histological features, as compared with cancer cell lines and animal models (Li J. et al., 2024). However, 3D cultures derived from CRC patients consist of phenotypically heterogeneous and interchangeable spheroid-forming cells and can therefore show different growth rates and drug sensitivity (Coppo et al., 2023).

Accumulating evidence suggests that CRC cells represent phenotypically dynamic (rather than static), heterogeneous cell populations that display cell plasticity characteristics. Moreover, organoids derived from CRC and metastatic tissues also showed preserved genetic diversity and morphological stability (Buzzelli et al., 2018). Tumor organoids have the ability to more accurately simulate the complex TME and can be utilized for screening anti-tumor drugs, predicting patient responses to drug therapy, and guiding personalized treatment plans (Qu et al., 2024).

The main advantages and limits of different pre-clinical *in vitro* models used in CRC are summarized in Table 1 (Reidy et al., 2021; Habanjar et al., 2021; Qu et al., 2024; Liu Y. et al., 2024). The strengths of 3D *in vitro* CRC models are extensively reported in this review, but weaknesses also exist. The primary challenges are the technical complexity and cost since the creation and maintenance of 3D cultures is more complex and expensive than traditional 2D models, limiting their utility in some settings. The scalability issues are also another problem because some 3D models may not easily scale for large-scale drug screening or clinical applications. Additionally, the limited standardization of protocols for *in vitro* 3D models leads to variability in results across different laboratory and research projects. Furthermore, despite the advancements, many 3D models fail to fully replicate the intricate TME since they are missing key components like blood vessels, stroma, immune cells, and the ECM.

**TABLE 1:** Comparison between the different pre-clinical models.

Model techniques	Advantages	Limitations
2D cultures	Easily established and maintained, low cost	Not representative, lack of relevant microenvironment
Spheroids	Easily scalable, allowing high-throughput drug screening	Lack of heterogeneity
PDOs	Capture phenotypic and genotypic features of the patient’s tumor, allowing for extensive and rapid *ex vivo* drug testing	Time consuming, can be expensive
Scaffold-based models	Imitating the natural microenvironment Natural hydrogels have a high biological relevance, synthetic hydrogels have a high reproducibility	Complexity in design and fabrication, limited reproducibility

## 3 The natural tumor microenvironment

**FIGURE 3:**
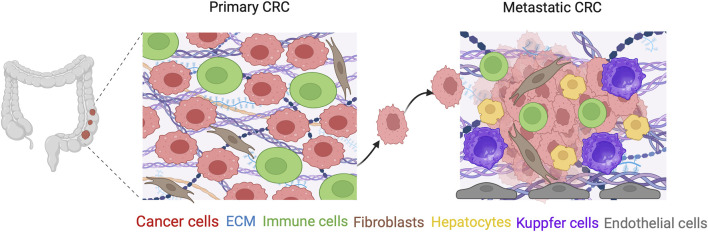
3D models mimicking the TME of CRC and mCRC compartments. A complex network of cellular and ECM microenvironmental interactions is critical for cancer progression, and is essential not only for the primary CRC tumor, but also for the metastatic scenario. Except for the immune and stromal cells, liver sinusoidal endothelial cells, Kupffer cells and other liver macrophages are necessary for maintaining the mCRC tissue environment in the liver. Created with BioRender.com.

Cancer research has expanded its focus to incorporate the importance of the TME, which is a dynamic ecosystem encompassing both cellular and acellular components (Palucka and Coussens Lisa, 2016). The cellular components of the TME are a heterogeneous population of cancerous and non-cancerous cells. Non-cancerous cells are mainly immune cells and stromal cells. The TME acellular compartment includes the ECM, growth factors, cytokines, and other soluble mediators (Walker et al., 2018). This complex network of interactions is critical for cancer progression and the response to therapy. The interaction of cancer cells with their microenvironment is essential not only for the primary CRC tumor but also for the metastatic scenario. The invasion of cancer cells from the primary tumor via the ECM of the stroma is a critical stage that is driven by a multitude of biochemical and biophysical cues in the acellular ECM (Peela et al., 2017). Upon entry of the circulating CRC cells from the blood to the liver, several basic steps are implicated in the formation of liver metastases. Hepatic stellate cells (HSCs) that are activated by the pro-inflammatory cascade play vital roles in liver physiology and fibrogenesis and produce ECM-related proteins (Tsilimigras et al., 2023; Chandra et al., 2021). Neo-vascularization is mediated by vascular endothelial growth factor (VEGF) produced by the tumor cells or activated liver-resident macrophage-like Kupffer cells (Figure 3). Oxygen and nutrients are thereby supplied to the tumor cells, and the metastatic cells can multiply and enlarge to form detectable tumors. Moreover, in hepatic mCRC, increased fructose metabolism was observed through the upregulation of the enzyme aldolase B (ALDOB), providing additional fuel for metastatic growth (Bu et al., 2018). Thus, as the metabolic hub of the entire organism, the liver appears to create a unique environment that allows or forces cancer cells to engage in certain metabolic activities for their colonization. In addition, oncogenic KRAS and transforming growth factor-beta (TGF-β) have been shown to promote and maintain CRC metastasis via the regulation of immunity and cell–cell contact (Boutin et al., 2017). Understanding the molecular mechanisms that enable metastasis, determining and validating the key targets that maintain metastasis, and identifying how these targets might also influence primary tumor maintenance are all critical knowledge gaps, that could be filled by the use of advanced *in vitro* 3D models.

### 3.1 The extracellular matrix in the tumor microenvironment

The ECM compartment regulates cancer cell behaviour such as proliferation, adhesion, migration, and differentiation (Bonnans et al., 2014) and its function in cancer and metastasis has been extensively discussed (Kai et al., 2019). The ECM constitutes over 300 components, the majority of which are proteins and glycoproteins (incl. collagens, laminins, elastin, fibronectin, and tenascins), proteoglycans (incl. heparan sulphate), and glycosaminoglycans (incl. HA). At the structural level, there are two distinct ECM compartments to consider: the interstitial compartment, which is a network mostly composed of fibrillar collagens and non-collagenous glycoproteins, and the basement membrane (BM), which separates the epithelial layer from the underlying tissue (Manou et al., 2019) (Nyström, 2021). The BM is made up of a fibril mesh mostly composed of type IV collagen and laminins. Laminins are composed of three polypeptides (α1-5, β1-4 and γ1-3) in diverse combinations, resulting in fifteen known variations forming a cross-like structure (Aumailley, 2013).

As tumor progression occurs, the cancer cells subsequently interact with a completely different niche, the tumor-associated stroma, which is composed of activated mesenchymal cells and a collagen I-rich ECM with a peculiar topography. Research has demonstrated that the ECM of CRC TME dynamically shifts during each stage of development, indicating its importance in cancer progression (Li ZL. et al., 2020). In addition to changes in the composition of the ECM, the stiffness of the ECM increases as CRC advances (Kawano et al., 2015), and increased stiffness may limit drug delivery and enhance resistance (Schrader et al., 2011). Studies on CRC have shown that changes in the ECM can either limit tumor development or promote tumor progression (Miura et al., 1993). These mechanical qualities emerge as fundamental drivers of cellular activity since, for example, increasing matrix stiffness can lead to an increase in focal adhesions, rupture of adherent junctions and accelerated proliferation (Paszek et al., 2005). Therefore, an *in vitro* CRC model including ECM, either incorporated into their architecture or provided by cells that release ECM components, would be beneficial.

Collagen has been used for 3D *in vitro* pharmacological testing (Magdeldin et al., 2014) or cell invasion (Nyga et al., 2013) with HT29 and HCT116 cell lines, as well as to stimulate morphological differentiation in some colon cell lines (Del et al., 1991). Collagen hydrogel cultures have also been utilized to explore the role of matrix-degrading metalloproteinases that are expressed in colorectal cells (Yamamoto et al., 2008). Typically, epithelial and mesenchymal cells interact with type IV collagen via receptors such as integrins. HA have also been found to be abundant in the ECM of CRC. Lue et al. used hydrogelsmade of hyaluronan and collagen I, to create a 3D matrix for the co-culture of CRC PDO and cancer associated fibroblasts (CAFs) (Luo et al., 2021). They discovered that these hydrogels could retain the critical molecular properties of the original patient tumors in CRC PDOs. Digging into the cellular compartment of TME is the next step after exploring its acellular components.

### 3.2 Stroma cells in the tumor microenvironment

**FIGURE 4:**
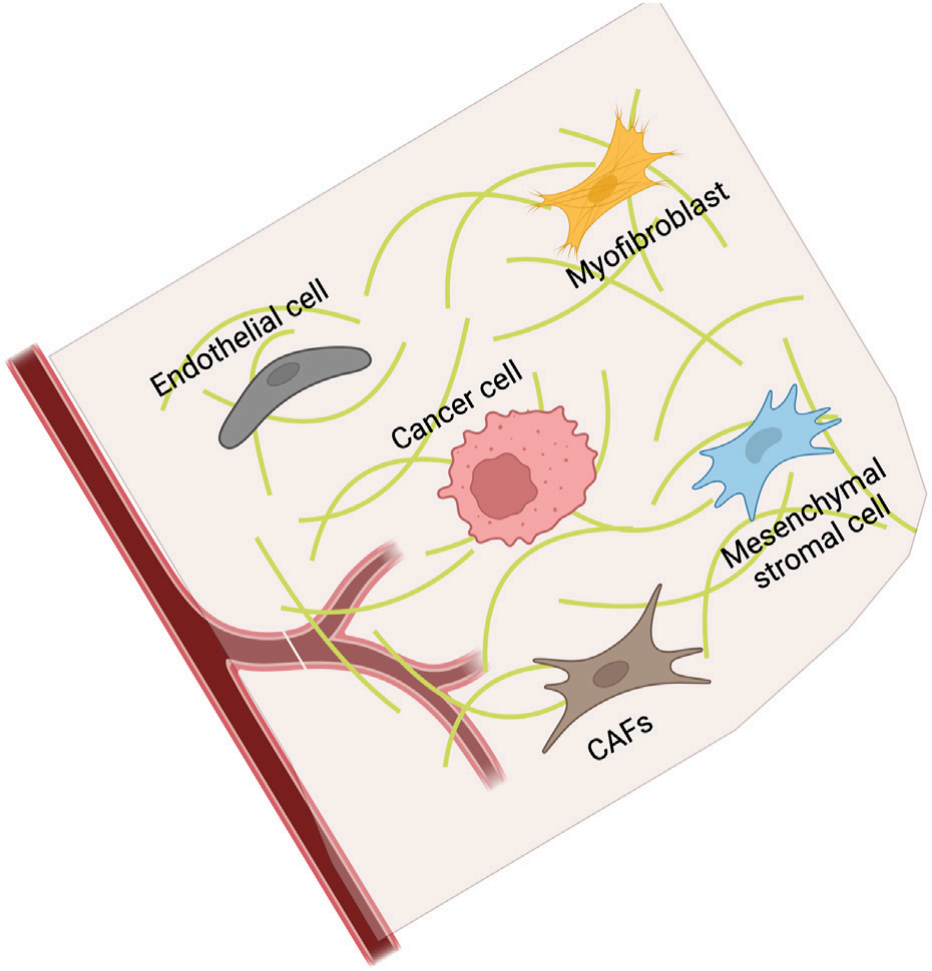
Stromal cell compartment is a complex key environment surrounding the cancer cells, consisting of MSCs, fibroblasts, and endothelial cells. Created with BioRender.com.

It is well-known that the TME of CRC is composed of both malignant and various non-cancerous cells. Stromal cells are a key component of the non-cancerous cells in the TME. It consists of several cell types including mesenchymal stromal cells (MSCs), fibroblasts, and endothelial cells (Figure 4). As a crucial part of the TME, the tumor stroma influences and contributes to cancer progression, metastasis and treatment resistance.

#### 3.2.1 Mesenchymal cells

In advanced CRC, the stroma contributes up to 50% of the primary tumor mass (Binnewies et al., 2018), with the majority of stromal cells being MSCs. A number of mesenchymal-derived cell types with similar characteristics are included, such as myofibroblasts and CAFs, and they can be resident cells or recruited to the tumor site to support development (Sai et al., 2019). CRC cells cultured in 3D using Matrigel with combinations of crypt growth factors such as EGF, Wnt, R-Spondin 1 and Noggin formed an invasive disc-like colonies (Ludwig et al., 2013). 3D CRC cultures remain responsive to growth factor stimulation and the invasive phenotype is reversible. CRC cells lose the epithelial phenotype and simultaneously gain the mesenchymal phenotype, undergoing epithelial-to-mesenchymal transition (EMT) that allows them to metastasize (Buhrmann et al., 2019). Furthermore, the TME were shown to influence the EMT of CRC cell lines when grown in 3D-alginate culture (Buhrmann et al., 2021). Indeed, EMT is a critical process in cancer metastasis which enhanced their invasiveness and mobility.

Moreover, CRC cells co-cultured with mesenchymal stromal cells and immune cells showed that the presence of mesenchymal cells in the TME promote an immunosuppressive environment (Leonard et al., 2021). Leonard et al. developed a 3D model of CRC that includes MSCs and immune cells to better mimic the TME and study its effects on both tumor growth and treatment response (Leonard et al., 2021). They used a gelatin-methacryloyl-based hydrogel culture system incorporating CRC cells, MSCs, and a monocyte cell line. The presence of stromal cells increased the transcription of matrix remodeling proteins (FN1 and MMP9) and the release of tumor-promoting immune molecules such as macrophage inhibitory factor (MIF), Serpin E1, chemokine (C-X-C motif) ligand 1 (CXCL1), interleukin-8 (IL-8) and chemokine (C-X-C motif) ligand 12 (CXCL12). Stromal cells altered the expression of immunotherapeutic targets on cancer cells, such as epidermal growth factor receptor (EGFR), Cluster of Differentiation 47 (CD47) and Programmed death-ligand 1 (PD-L1). Treatment with an EGFR inhibitor (PD153035) showed altered PD-L1 expression in CRC cells, but only in the absence of MSCs.

#### 3.2.2 Cancer-associated fibroblasts

CRC development involves dynamic interaction among malignant cells, components of the microenvironment (stromal and vascular endothelial cells), and the immune system (Mascaux et al., 2019). Several cell types from the TME can be recruited in the cancer progression and become recruited to fulfill critical pro-tumorigenic roles. This enables new cancer cells to elude immune system identification and establish a niche in which to grow unchecked (de Visser and Joyce, 2023). Fibroblasts are among the first cell types recruited within the TME, and they undergo a process known as activation when the crosstalk with cancer cells begins. Studies using 3D interpenetrating networks of collagen and alginate have shown that CAFs can switch between inflammatory and myofibroblastic states, which affect their role in tumor progression (Cao et al., 2021). The ECM is mainly produced and organized by fibroblasts, which serve as a structural scaffold for tissue architecture as well as a reservoir for growth factors and cytokines that can be released during matrix remodeling and cleavage (Arroyo and Iruela-Arispe, 2010). These cytokines and growth factors can influence the onset and course of CRC (Crotti et al., 2017). High stromal infiltration has been shown to be related to shorter overall survival in CRC patients. This is because both MSCs and CAFs secrete growth factors such as fibroblast growth factor (FGF) or hepatocyte growth factor, which can promote tumor growth (Turley et al., 2015), as well as TGF-β which fosters metastasis and leads to an immunosuppressive environment (Tauriello et al., 2018). Cao et al. showed the dual roles of CAFs in promoting and inhibiting tumor growth by using a 3D interpenetrating network of collagen and alginate (Cao et al., 2021). They found that CAFs can switch between the inflammatory-state having an anti-tumorigenic effect to the EMT-promoting myofibroblastic-state depending on the mechanical properties of the 3D environment. Understanding the conditions that cause CAFs to switch states can help in developing targeted therapies for cancer treatment by either promoting the anti-tumorigenic properties or inhibiting the pro-tumorigenic functions. Micalet et al. investigated how CAFs from different CRC patients affect the TME, particularly focusing on tissue stiffness (Micalet et al., 2024). Different patient-derived CAFs showed varied abilities to remodel the ECM where some stiffened the matrix through active contraction, while others softened it via enzymatic activity.

#### 3.2.3 Endothelial cells

Cell heterogeneity within a 3D model is an advanced concept for evaluating drug efficacy, understanding chemoresistance and developing TME-targeted therapies. In one study, a 3D multicellular model for CRC was constructed using the CRC cell line SW480 with CAFs and endothelial cells, showing improved expression of several tumor-related genes including *IL1B, FCGR2A, FCGR3A, CYBB, SPI1, CCL2, ITGAM*, and *ITGB2* (Wang et al., 2023). Other studies have reported increased drug resistance when co-culturing CRC cell lines with CAFs and endothelial cells (Zoetemelk et al., 2019). Briefly, they used HCT116, SW620 and DLD1 cells, which were co-cultured with fibroblasts and endothelial cells in 3D spheroids. Treatment was performed with 5-fluorouracil (5−FU), regorafenib and erlotinib. The results showed a dose-dependent increase of erlotinib sensitivity. Interestingly, simple 3D spheroid and 3D co-cultures responded distinctly to drug treatment, and the signalling pathways were also regulated differently. Moreover, Chen et al. have developed a 3D printed *in vitro* model which mimics the TME by co-culturing CRC cell lines, CAFs, and tumor-associated endothelial cells in 3D-printed scaffolds (Chen et al., 2020). This 3D model exhibited physiological activity with high drug resistance. To preserve the complex TME of CRC, freshly excised CRC samples were fragmented and placed between collagen type I sponges in a “sandwich-like” format and then cultured in a perfusion bioreactor (Manfredonia et al., 2019). This approach shows promise for more accurate *in vitro* models which can predict patients’ responses to treatments, potentially guiding therapeutic decisions more effectively. Therefore, compared with a single-cell model, the use of a 3D multicellular co-culture models is indeed a better option for mimicking the CRC environment.

### 3.3 Immune cells in the tumor microenvironment

Immune cells are additional major cell types following stromal cells in the TME. The immune infiltrate in CRC varies by subtype and may include T cells, B cells, neutrophils, monocytes, macrophages, natural killer (NK) cells and mast cells (Zhang et al., 2018). Under normal conditions, the immune system can eliminate cancer cells. Tumors have devised strategies to overcome this by polarizing immune cells into tumor-promoting phenotypes (Heichler et al., 2020).

**FIGURE 5:**
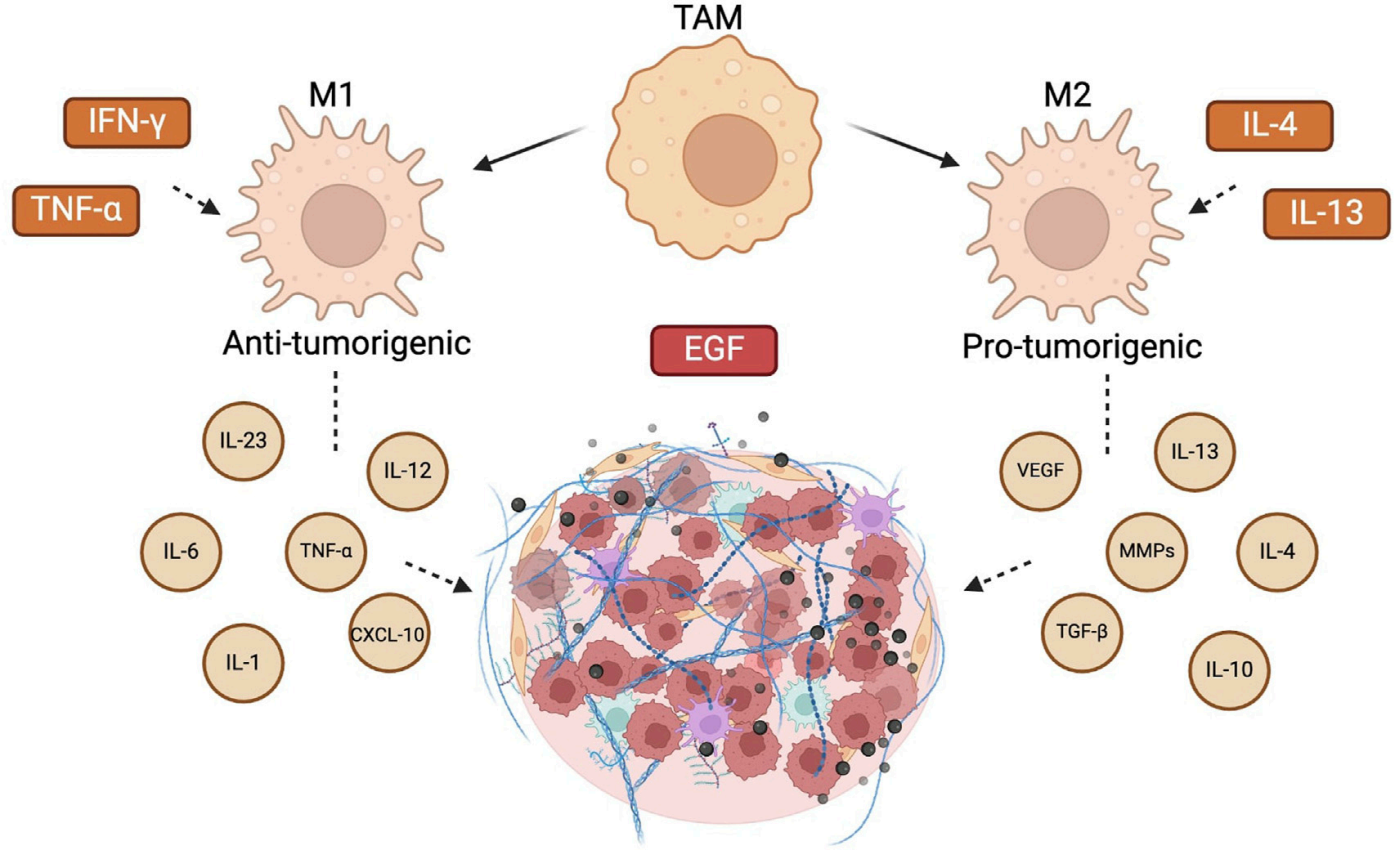
Interaction between CRC and TAMs. Different stimuli can polarize macrophages into two basic types, anti-tumorigenic M1 and pro-tumorigenic M2 phenotypes. M1 macrophages can be stimulated by interferon IFN-γ and express inflammatory factors including interleukin IL-1β, IL-6, and tumor necrosis factor TNF-α. M2 macrophages are stimulated by IL-4 and IL-13 and express IL-10 and transforming growth factor TGF-β, among other cytokines. Created with BioRender.com.

Dynamic and heterogeneous interactions between tumor cells and the surrounding microenvironment fuel the occurrence, progression, invasion, and metastasis of solid tumors. The TME plays a key role in modifying the plasticity of tumor cells and offers immunological escape strategies. Tumor-associated macrophages (TAMs) play a pivotal role in this process (Figure 5). Numerous studies have shown that TAMs can cause genetic instability in cancer cells (Bonavita et al., 2015), induce angiogenesis, increase tumor development and contribute to ECM degradation (Guerriero, 2018). TAMs can differentiate into pro-inflammatory anti-tumorigenic M1 and anti-inflammatory, pro-tumorigenic M2 phenotype. M1 are stimulated by TNF-α and IFN-γ to produce pro-inflammatory cytokines such as IL-1, IL-6, IL-12, IL-23, CXCL-10 and TNFα and in that way contribute to destroying tumor cells in the TME. The M2 phenotypes are, on the other hand, stimulated by IL-4 and IL-13 to produce and secrete growth factors such as VEGF and TGF-β, MMPs and other cytokines such as IL-10, IL-13 and IL-4, and in that way contribute to increased proliferation of tumor cells and angiogenesis in the TME. Depending on which chemokines, cytokines and growth factors are present in the TME these two types of macrophages can transform into each other, which is important for CRC progression and metastasis (Hou et al., 2024). For instance, STAT3 has been shown to be the main transcription factor for TAM transformation (Xia et al., 2023) and inhibition of the STAT3 signaling leads to reduced CRC metastasis (Zhang et al., 2022).

Immune cells in the TME are represented by the diversity of cells including TAMs, dendritic cells, regulatory T cells (Tregs), cytotoxic T cells (CD8^+^ T cells), and myeloid-derived suppressor cells (MDSCs) (Joyce and Pollard, 2009). Depending on the tissue context and the presence of signaling molecules, these cells could either promote or suppress tumor progression, contributing to immune modulation in cancer. A study of the TME from left-sided colon cancer and right-sided colon cancer based on scores from TCGA revealed that there was a difference in TME profiles (Liu D. et al., 2024). Left-sided colon cancer had high scores of non-differentiated M0 macrophages, activated CD4^+^ T cells, dendritic cells, NK cells and monocytes. On the other hand, right-sided colon cancer had higher scores of inflammatory M1 macrophages, neutrophils and CD8^+^ T cells resulting in a worse prognosis, especially for advanced stage (III/IV) colon cancer. Right-sided colon cancer showed elevated expression of programmed cell death protein-1 (PD-1), suggesting that these patients might respond better to treatments with immune checkpoint inhibitors (Liu D. et al., 2024). Numerous studies have shown that TAMs can cause genic instability in cancer cells (Bonavita et al., 2015), induce angiogenesis, increase tumor development and contribute to ECM degradation (Guerriero, 2018).

TAMs also produce EGF which can increase cancer cell migration and invasion by binding to EGFR expressed on cancer cells (Zhao et al., 2016). Macrophages are involved in regulation of collagen fibers and might thus promote cancer migration towards blood vessels since cancer cells use collagen for migration (Joyce and Pollard, 2009). PD-L1 is expressed by cancer cells, dendritic cells, B cells as well as TAMs, indicating pro-tumorigenic function which complicates clarification of the mechanism behind the anti-PD-L1 therapies (Du et al., 2024; Leonard et al., 2024). Strasser et al. showed that the TME of CRC consists of both pro- and anti-inflammatory immune cells which promotes progression of the CRC and might explain why inhibition of PD-1/PD-L1 is not that effective in CRC (Strasser et al., 2019). Three CRC cell line-based models of HCT-116, an *in vitro* 2D model, an *in vitro* 3D model of spheroids and an *in vivo* xenograft mice model were used to test the effect of sequential micro-immunotherapy medicine on M1 and M2 macrophages. This study suggests that 3D spheroid models provide better understanding and analysis of drug response (Jacques et al., 2022).

Tregs are either circulating or tissue-localized cells expressing a variety of inhibitory proteins that serve as control of immune safety. With other cells present in TME such as M2 macrophages, tolerogenic dendritic cells, and myeloid-derived suppressor cells, Treg cells can maintain the immune equilibrium by controlling function of different T cells by expressing a variety of proteins such as PD-1, lymphocyte activation gene-3 (LAG3) and cytotoxic T-lymphocyte antigen-4 (CTLA-4). Tregs are induced and differentiated by traditional T cells and are suggested to have the ability to inhibit activation and differentiation of CD4^+^ helper T cells and CD8^+^ cytotoxic T cells. However, Tregs also appear to stop the host´s anti-tumor response and enhance tumor immune escape, growth and metastasis (Verma et al., 2022; Li C. et al., 2020). This pro-tumor property of Tregs is due to the secretion of molecules (TGF-β, IL-10, and IL-35) which regulate the expression of inhibitory receptors. Since Tregs cannot be eliminated from the tissue, approaches to controlling actions of Treg cells have been proposed in form of immune checkpoint inhibitors (Li C. et al., 2020). Since immunotherapies still fail to gain CRC survival, new functional assays and cancer cell models are required. Natural killer (NK) cells belong to innate lymphoid cells and have the capacity to kill tumor cells by releasing cytotoxic molecules and by producing cytokines and chemokines that recruit other immune cells such as dendritic cells (Marchalot and Mjösberg, 2022). However, Mao et al. showed that NK cells exhibited tumor-activating characteristics when they were co-cultured with tumor cells thus promoting progression of colon cancer liver metastasis (Mao et al., 2024). Courau et al. have showed that co-culture of CRC cell lines (HT-29 and DLD1) together with T and NK cells in a 3D spheroid model is a good model of studying tumor-lymphocyte interactions and immunotherapy (Courau et al., 2019).

## 4 Models for treatment of CRC and mCRC

Preclinical cancer research entails the testing of novel drugs in non-human subjects, necessitating several experiments based mostly on 2D-cultured cancer cell lines and animal experiments. For decades, *in vivo* models have been regarded as the gold standard for oncological research to investigate the complex multicellular components of tumors (Tosca et al., 2023).

The research on the TME has recently been gaining attention due to its important role in tumor growth, progression, and response to therapy. Because of this, the development of 3D *in vitro* cancer models that mimic the interactions in the TME, the tumor structure and complexity is of great relevance for drug development. The antimetabolite 5-FU remains a crucial treatment strategy for CRC and the use in combination with folinic acid was shown to be a better outcome for patients (Petrioli et al., 1995). The incorporation of oxaliplatin has shown additional survival benefits (Wolpin et al., 2007). In metastatic disease, treatment strategies have become more complex, involving various combinations of chemotherapy FOLFIRI, (fluorouracil, folinic acid and irinotecan); FOLFOX, (fluorouracil, folinic acid and oxaliplatin); FOLFOXIRI, (fluorouracil, folinic acid, oxaliplatin and irinotecan) and XELOX, (capecitabine plus oxaliplatin). Results of molecular research have demonstrated the need to profile each mCRC for different mutations. Targeted therapies have further expanded the treatment options, but novel therapies still need to be developed (Aparicio et al., 2020). Multiple clinical trials have shown that tumors with activating mutation do not benefit from targeted therapies (Aparicio et al., 2020).

Even though survival rates have increased with the introduction of targeted therapies, one of the major challenges in treating CRC is the development of treatment resistance, especially in metastatic cases. CRC is a genetically diverse disease and new treatment strategies are needed, such as improved targeted therapies and immunotherapy. Immunotherapy is clinically used and FDA-approved for patients with high MSI, MSI-H, or mismatch repair-deficient (dMMR) CRC. These tumors have numerous mutations that make them more visible to the immune system. The dMMR/MSI-H cancers are associated with strong lymphocytic infiltration in and around the tumor (André et al., 2020). Immune checkpoint inhibitors (ICIs) have as a consequence emerged as a promising immunotherapy for CRC patients with this type of mutations (Passardi et al., 2017; Hirano et al., 2021). These therapies aim to enhance the immune system’s ability to attack tumor cells by blocking the proteins responsible for inhibiting the immune response, such as PD-1 and CTLA-4. However, microsatellite stable (MSS) CRC, which represents the majority of cases, has shown limited response to ICIs since MSS tumors often have low levels of lymphocytic infiltration and low levels of immune checkpoint molecules (Chen et al., 2021). Ongoing research focuses on combining ICIs with other immunotherapies or conventional treatments to enhance efficacy in MSS CRC (Makaremi et al., 2021).

Cell growth in 3D settings not only promotes phenotypic changes in cell morphology, sensitivity to stimuli, cell activities and gene expression patterns, but it also affects the response to therapeutic agents (Romero-López et al., 2017). Indeed, both normal and cancerous cells retain their distinct behaviors in the body owing to their 3D environment, which includes heterogeneous and dynamic cell-cell and cell-matrix interactions (Kessenbrock et al., 2010). Hachey et al. showed that when CRC cell gene expression, heterogeneity, growth, and responsiveness to standard chemotherapy were examined, cells growing in vascular micro-tumors (VMT) more closely resembled those growing as *in vivo* murine xenografts (Hachey et al., 2021). It is likely that 3D cell culture models have demonstrated exceptional capabilities in bridging the gap between oversimplified 2D systems and subsequent animal experiments for safety assessment prior to clinical trials. Increasing the predictive capacity of 3D culture models may help to minimize the number of animals used by the pharmaceutical industry for drug efficacy and toxicity testing. 3D cell cultures also demonstrated stronger stability and longer life spans than 2D cell cultures did, making them better suited for long-term studies on cell interactions or drug effects. Similarly, the development of patient-derived cells in such 3D cultures may provide unparalleled opportunities for personalized pharmacogenetic techniques that animal models cannot address.

By using Kras mutant mouse models of CRC, it was reported that wild-type Kras plays a substantial role in the fitness, evolution, and treatment susceptibility of Kras mutant cells *in vivo* (Najumudeen et al., 2024). The combination chemotherapy regimen based on oxaliplatin remains the first-line treatment for CRC, notably including the FOLFOX regimen and the XELOX regimen. However, CRC becomes resistant to oxaliplatin, and the underlying mechanism is unknown (Deng et al., 2024). Deng et al. established a CRC subcutaneous tumor model and metastasis model via oxaliplatin induction to generate human CRC drug-resistant cell lines (Deng et al., 2024). They demonstrated that increased H3K79 methylation may be responsible for the resistance of CRC cells to oxaliplatin.

Inhibiting HIF-1α can improve the CRC microenvironments and increase treatment sensitivity (Su et al., 2024). Knocking down HIF-1α or its upstream regulator BIRC2 in animal experiments leads to tumor growth inhibition by increasing CD8^+^ T cell infiltration. Moreover, bleomycin or doxorubicin had greater anti-tumor effect *in vivo* when HIF-1α was knocked down. Notably, the TGF-β produced by cancer cells and CAFs is central to immune suppression within the TME and contributes to tumor immune evasion and poor responses to cancer immuno-therapy (Batlle and Massagué, 2019). TGF-β released in the TME attracts fibroblasts and induces the production of CAFs (Lebrun, 2012). Additionally, TGF-β promotes immune evasion in genetically reconstituted mCRC (Tauriello et al., 2018). In mCRC mouse models, Li et al. detected a decrease in CAFs through the TGF-β receptor inhibitor SB525334, which sensitized mCRC to immunotherapy by improving the tumor immune microenvironment and led to significantly reduced tumor uptakes of 68Ga-FAPI by PET/CT (Li K. et al., 2024). The ^68^Ga-FAPI PET/CT imaging helps select patients with mCRC who can benefit from immunotherapy and guides the precise timing of TGF-β inhibition to optimize the combination strategy with immunotherapy.

Many studies illustrated the efficiency of 3D *in vitro* models to replace animal models in CRC research. Vlachogiannis et al. developed a biobank of PDOs from metastatic, heavily-pretreated colorectal and gastroesophageal cancer patients, to assess molecular profiles and drug responses. PDOs were derived from sequential biopsies taken at different stages including baseline, at the time of best response and at disease progression, as well as from multiple tumor sites. They tested 19 PDOs in a screening using a library of 55 drugs currently in phase I-III clinical trials or in clinical practice. By comparing the drug responses of PDOs and PDO-based orthotopic mouse tumor xenografts with patient responses in clinical trials, they concluded that PDOs effectively reflect patient heterogeneity and can be a valuable tool for predicting the response of specific therapies. It was also proved by Svanström et al. that the environment in a 3D culture model more closely resembles the native tissue. They showed that cells cultivated in both patient-derived and 3D printed scaffolds responded similarly to hypoxic circumstances, which is a significant feature in tumors. Moreover, Chen et al. co-cultured CRC and CAFs in 3D printed scaffolds to mimic ECM. The model showed a physiological activity similar to *in vivo* tumors, with high drug resistance and overexpression of tumor-related markers.

The patient-derived xenograft (PDX) model is an effective tool for studying tumors because it can maintain 3D cell-cell interactions and capture tumor heterogeneity (Drost and Clevers, 2018). Tumor-derived organoids in culture may accurately reflect the gene expression patterns, gene regulatory networks, point mutations, and DNA methylation patterns of tumor cells *in vivo* (Wang et al., 2022). Normal-tissue-derived organoids retain the normal genomic features of normal epithelial cells *in vivo*. Recently, established 3D organoid culture technologies have transformed cancer research by enabling more realistic and scalable replications of both tumor and microenvironmental structures (Drost and Clevers, 2018; Tuveson and Clevers, 2019). It can serve as an appropriate preclinical model for the evaluation of high-throughput drug response screening and the mechanistic studies of tumorigenesis, invasiveness, and metastasis (van de Wetering et al., 2015). Organoid-based 3D models have been proposed as a bridge between *in vitro* and *in vivo* models (Kim et al., 2020). Additionally, the CRC organoid orthotopic transplantation technique and a genetically engineered autochthonous mouse adenoma model were used to conduct a comprehensive epigenomic and transcriptomic analyses to determine the variables required for tumor initiation in these cancers (Goto et al., 2024). This study investigated how naive colon cancer organoids produced *in vitro* with *Apc*-null, *Kras* ^*G12D*^ and *Trp53*-null (AKP) mutations acclimated to the *in vivo* native colonic environment. These findings revealed that SOX17, a transcription factor involved in endoderm and fetal foregut development (Spence et al., 2009), is required for the growth of adenomas and CRC. Importantly, organoid models have limitations due to closed cystic structure instead of an *in vivo*-like apically open architecture (Sato et al., 2009), short lifespan that requires breaking up the culture every few days for passaging (Nikolaev et al., 2020), and lack of topobiological stability and consistency owing to stochastic growth in 3D matrices (Sato et al., 2009).

## 5 Nanotools for diagnostic and therapeutic applications of 3D models and their ECM components

Several excellent reviews focusing on the nano- and microengineering of the TME for different diseases already exist (Jin et al., 2020; Kim et al., 2023; Chen et al., 2018; Song et al., 2018). Cancer diagnostics and therapeutics can improve significantly by using nanotools for imaging and treatment applications (Jin et al., 2020). The collection of nanoscale tools discussed in a review by Jin et al. includes quantum dots, nanoshells, gold nanoparticles (NP), liposomes, carbon nanotubes, polymeric micelles and dendrimers. Moreover, recently described engineered therapeutic models involve a variety of 3D model scaffold systems such as electrospun nanofibers, nanoprinted scaffolds, self-assembled peptide hydrogels, and systems incorporating dynamic physiological features into both nano- and microstructure substrates for cancer research (Kim et al., 2023; Chen et al., 2018). Electrospun nanofibers with the size of collagen fibrils, or surface modified with ECM-derived proteins were preferable (Chen et al., 2018). Drug screening platforms using micro- or nanoengineered technologies are in clinical use. Patient-derived tissue slices on microfluidic devices were tested for multiplexed drugs in both glioblastoma and metastatic CRC. Referring to clinical trials, Kim et al. mention that patient-derived organoids-on-a-chip were tested to screen drugs and assessing response to guide CRC treatment of the patients (Kim et al., 2023; Li et al., 2011). Moreover, tissues have been employed to establish a 3D bio-printed model and to predict clinical efficacy of chemotherapeutic drugs of CRC patients. Song et al. describe advanced modern biomicroscopic tools with commonly used methods and materials to engineer 3-D cancer or other disease models (Song et al., 2018). A fabricated 3D cell-mimicking hydrogel microtissue with a photocurable hydrogel as the ECM could control the cell size as well as the cell type (Li et al., 2011). In another example, a method to encapsulate single cells within 3D matrix structures using photopolymerization technique in hydrogel micro-niches was described to allow both cell adhesion and nutritional permeability (Bao et al., 2017). A study by Yan et al. revealed that a novel supramolecular up-conversion NP with incorporated platinum (IV) oxaliplatin prodrug and a polymer with inhibition capacity for a certain bacteria existing in the community of CRC, as well as with polyethylene glycol-azobenzene could together enhance drug-loading and enable on-demand drug release for drug-resistant CRC treatment (Yan et al., 2024). This study indicates that multiple dynamic chemical designs integrating drug loading and release of a single system provide a promising candidate for precision therapy.

Preclinical studies indicate that TAMs represent an attractive target for cancer therapeutics (Miao and Huang, 2015). TAMs express high amounts of the mannose receptor, which has been used as a targeting ligand for NP-based TAMs delivery (Zhu et al., 2013). With better knowledge about M1 macrophages having antitumorigenic properties, focus of NP development has shifted from exclusively depleting and imaging all TAMs to modulating the ratio of M1/M2 macrophages for improved therapy (Miao and Huang, 2015). CAFs secrete diverse growth factors, cytokines, and extracellular matrix (ECM) proteins that fuel tumor growth and progression. To specifically inhibit CAF activation, proliferation, or function, small-molecule inhibitors, antibodies, or NPs can be tailored for therapeutics (Shah et al., 2025). However, considerations must be taken on any potential adverse effects on normal fibroblasts.

### 5.1 Modulating the stroma of the tumor

The modulation of stromal cellular components such as endothelial cells, CAFs or TAMs, by small molecules or nanodrugs can facilitate the remodeling of tumor blood vessels or ECM, are recognized as major components that promote cancer progression, therapy resistance, and metastasis formation. Miao et al. highlight in their review that this can be performed by distribution of NPs to these cellular components in stroma (Miao and Huang, 2015). Major components of type IV collagen, laminin, entactin (nidogen) and fibronectin are building up the vasculature basement membrane. The higher tumor vasculature compared to healthy vasculature leads to leaky and loosely compacted vasculature, which tends to be abnormally permeable to macromolecules and NP (10–100 nm, in diameter). Stroma cells, on the other hand, can compress vessels and inhibit NP penetration. To improve drug penetration, proteases including hyaluronidase, collagenase, matrix MMP-1 and MMP-8 are frequently used to decrease the level of NP-limiting tumor glycosaminoglycans (Miao and Huang, 2015). Another approach reviewed by Miao et al. was depleting HA by using PEGylated human recombinant PH20 hyaluronidase (PEGPH20). This was shown to increase macromolecule permeability and augment chemotherapy responses in a pancreatic model. Inhibiting the enzyme critical for the stabilization of collagen networks, LOX, is another strategy (Liu et al., 2025). Findings highlighting the challenge of targeting ECM synthesis in cancer therapy are for example, studies using the combination of the LOXL2 antibody Simtuzumab with FOLFIRI chemotherapy or gemcitabine, which did not show significant clinical benefit in 280 patients with pancreatic cancer or CRC as reviewed by Liu et al. (2025).

## 6 Discussion

The use of animal models is both economically and ethically costly. Thus, there is the universal commitment that animal use in research should be reduced, replaced, and refined, known as the 3R principle established by Russell and Burch in 1959. According to the 3R principle, animal experiments should be replaced with alternatives whenever possible. Additionally, irreproducibility in scientific research has become a major issue. Despite the need for rigor when writing a scientific paper, more than half of publications are regarded as non-reproducible. In the context of animal research, selecting an appropriate experimental model within the 3R concept is crucial, as irreproducibility causes an annual loss of 28 billion dollars in biomedical research without fruitful outcomes. The expansion of the 3R to the 5R, by introducing “robustness” and “reproducibility”, may provide a framework for research improvement. There is a significant lack of data specifications in scientific articles, revealing gaps in the presentation of critical details, such as environmental conditions and experimental protocols. Certainly, one of the advantages of 3D models is their ability to simulate the biological TME in the laboratory. From this perspective, reproducibility is extremely beneficial, as is the ability to standardize experiments.

Soon, the utilization of 3D *in vitro* cancer models may become a required step between 2D *in vitro* and *in vivo* animal models. Identifying and eliminating those drugs that have no interesting efficacy in 3D *in vitro* cultures will reduce animal use as well as the associated costs and ethical issues. This could increase the number of effective drug candidates that have advanced to clinical development, thereby lowering the number of patients who receive ineffective treatments and increasing the success rate of clinical trials.

Additionally, *in vivo* model has several technical constraints such as the number of animals required for time-point experiments and the sensitivity of cell imaging *in vivo*. Another drawback is that species differences may impair the clinical translation of results. Moreover, animal models are both time-consuming and require expensive commitments, not to mention the ethical concerns. The need for precise and trustworthy results has driven the development of more biomimetic and clinically relevant disease models, to prevent early clinical trials and save time and resources. Various *in vitro* models have been published and used to assess potential drug candidates, which are often examined in 2D cell monocultures. Only 5% of drugs proven to be active in such cell culture models have reached the clinical trials (Kola and Landis, 2004; Ibarrola-Villava et al., 2018). Current evidence shows that 3D culture models may better capture the mechanisms of drug resistance identified in tumors. 3D *in vitro* models can be very efficient in term of multiple drugs evaluation as shown in the study by Kondo et al. with a large-scale screening of 2,427 compounds on CRC organoids to uncover numerous “hit” drugs (Kondo et al., 2019). To solve the time-consuming process of PDO derivation and expansion, Ding et al. developed an automated microfluidics droplet platform that can construct patient-derived microorganospheres (MOS), allowing for quick clinical high-throughput drug selection in a substantially shorter period (Ding et al., 2022). By analysing samples from eight metastatic CRC patients, the authors revealed that studies using MOS can provide results within 7–14 days of biopsy collection, with drug responses consistent with clinical outcomes. Notably, the original tumor’s stromal and immune cells were maintained in the MOS, making it an effective tool for testing immunotherapies.

The advances in understanding cancer disease mechanisms related to TME will lead to a major revolution in the research field. However, the magnitude of this impact is determined by the effectiveness with which biomedical research breakthroughs are converted into clinical practice. This translation process entails the development of new drugs, of medical equipment, and of clinical procedures. According to National Institutes of Health (NIH), bringing a new drug or medical device from development to market takes on average 14 years and $2 billion (Health NIo, 2015). Therefore, we concur that laboratory results from 3D CRC models have a long way to go to clinical use. The challenges to translating those findings into clinical practice are well documented for each 3D approach (Bose et al., 2022; Price et al., 2023; Peng et al., 2025).

3D *in vitro* models of CRC own numerous potentials for guiding clinical decisions through several key mechanisms. Like in personalized drug screening, PDO 3D models can be generated immediately from tumor biopsies. These models keep the original tumor’s genetic, molecular, and histological properties, allowing clinicians to evaluate multiple therapeutic alternatives *in vitro* before deciding the most effective regimen for the patient (Thorel et al., 2024). This personalized approach can reduce trial-and-error prescribing while improving outcomes by identifying treatments that are more likely to succeed. In prediction of drug resistance, 3D models that can simulate the complexity of CRC TME, including ECM stiffness and cell heterogeneity, are valuable factors since those are known to influence drug resistance in CRC (Liu et al., 2020). Understanding how cancer cells interact with these factors in a controlled 3D environment helps clinicians to anticipate resistance mechanisms and adjust therapy accordingly, potentially combining treatments to overcome resistance. Additionally, while determining whether a drug works, 3D *in vitro* models enable researchers and clinicians to test different dosing regimens, combinations, and sequences of therapies in a milieu that mimics tumor physiology. This provides more information about treatment strategies, potentially reducing side effects and increasing efficacy. Furthermore, the benefit of quick testing in clinical timeframes since advances in bioengineering and culture techniques have reduced the time required to grow and test PDO 3D models, making it increasingly feasible to incorporate results into clinical timelines. For example, mini-organoids can sometimes be tested within 1–2 weeks, offering relevant data for oncologists treating aggressive or late-stage CRC (Zhou et al., 2021).

## 7 Conclusion

3D *in vitro* model systems that mimic the complex nature of the TME allow studies of cellular and molecular interactions offering valuable insights that would be challenging to obtain solely from clinical samples alone. There have been recent advancements in spheroid models and PDOs enhancing the understanding of the heterogeneic physiology of the TME. Emphasis on systematically including stromal cells, immune cells and endothelial cells in the 3D culture models, while analyzing these systems under defined experimental conditions will further improve the testing the effects of therapeutic agents and reliable outcome.

This review highlights the need for of validated *in vitro* models in cancer research. Today’s animal testing must be substituted with standardized 3D models including both cellular and acellular ECM. Establishing a 3D *in vitro* model in the cancer research area which considers the TME condition as much as possible could turn this fiction into reality. Furthermore, recent improvements in 3D culture design and co-culture techniques, will pave the way for the creation of real TME model that includes an increasing number of cell types. Combining these models with biological scaffold and ethical animal-free system is what we suggest for the future of CRC 3D *in vitro* models.

Recent advancements in technology are paving the way for the development of high-throughput 3D tumor model screening platforms. Combining multiple treatment modalities and nanotechnology that target different components of the TME holds promise for improving therapeutic outcomes. Multiplexed immunofluorescence and spatial transcriptomics are examples of novel methodologies that reveal detailed insights in the cellular composition and spatial organization of the TME. These approaches enable the identification of unique TME signatures associated with therapy response and resistance, guiding the development of personalized treatment strategies.

While 3D models could offer considerable advantages as previously discussed, their practical application in clinical settings remains limited. Researchers encounter several hurdles in bridging the gap between laboratory findings and real-world clinical use. One challenge is the lack of model standardization, as there is currently no universally agreed protocol to generate 3D *in vitro* models. Therefore, variability might lead to inconsistent data across laboratories. This lack of uniformity hinders reproducibility and complicates cross-study comparisons, which are essential for clinical validation. Moreover, not all patient tumors yield viable organoids, particularly in cases of advanced CRC or treatment-resistant CRC.

Nevertheless, even when reliable data is collected from 3D models, incorporating it into existing clinical decision-making pathways is difficult. Model-derived knowledge must be included into the decision-support system; otherwise, valuable laboratory findings remain underutilized. To increase the clinical utility of CRC 3D *in vitro* models, international guidelines or consensus protocols for culturing and analyzing 3D models must be in place. Encouraging open-source platforms for sharing validated techniques, including materials used, culture periods, and response evaluation measures, will benefit both researchers and clinicians. In addition, miniaturized assay formats (e.g., 96- well plate) optimized for 3D models could reduce reagent costs and increase throughput. Finally, training clinicians via interdisciplinary workshops and continuing medical education courses in precision oncology and 3D model interpretation might be a possible way.
